# Utilization of the Revised American College of Rheumatology (rACR) Scoring to Avoid Unnecessary Temporal Artery Biopsies—A Case Series

**DOI:** 10.3390/medsci10010011

**Published:** 2022-02-08

**Authors:** Tayyaub Mansoor, Noel P. Lynch, Hicham Rifai, Sean Hamlin, Darragh Moneley

**Affiliations:** 1 Royal College of Surgeons, D02YN77 Dublin, Ireland; noellynch@rcsi.ie (N.P.L.); seanhamlin@rcsi.ie (S.H.); 2 Department of Mathematical Sciences, Technological University, D07EWV4 Dublin, Ireland; rifai.hicham@gmail.com; 3 Vascular Surgery Department, Beaumont Hospital, D09V2N0 Dublin, Ireland; dmoneley@gmail.com

**Keywords:** giant cell arteritis, temporal arteries, biopsy

## Abstract

Introduction: The American College of Rheumatology (ACR) criteria, and more recently the revised ACR criteria (rACR), are a scoring system developed to aid in the diagnosis of giant cell arteritis (GCA). Our aim was to investigate the value of the non-biopsy criteria of the original ACR criteria and rACR criteria to predict GCA, and investigate the utilization of such scores to avoid biopsy when a very high or very low likelihood of a positive temporal artery biopsy TAB was predicted. Method: We conducted a retrospective cohort study of 59 patients undergoing TAB from 2013 to 2017 in Beaumont Hospital, a tertiary referral centre in Dublin, Ireland. Demographic data, biochemical results, presenting features, and histology results were collected and collated. Results: Data were analysed from 53 patients and ACR scores were compiled. Seventeen scored < 3 and thirty-six scored 3–5. All 11 positive biopsies were in the 3–5 score range. Forty-five patients were analysed with rACR scores. Eight were excluded due to not meeting the inclusion criteria. Of the 11 positive biopsies, 2 were in the 3–4 score range, and 9 were in the ≥5 score range. In the ACR method, 36% of all biopsies scored as low-risk pre-biopsy. In the rACR method, 84.4% of all biopsies scored in the low- and intermediate-risk group pre-biopsy and 15.6% of all biopsies scored in the high-risk group pre-biopsy. Conclusions: This study illustrates the potential value of the rACR scoring system as a useful tool to categorize patients according to risk with a view to avoiding unnecessary TAB. The data suggest that a TAB has a helpful role in low- and intermediate-risk groups but is of minimal benefit in the high-risk group.

## 1. Introduction

Giant cell arteritis (GCA) is large- and medium-sized vessel vasculitis that has a reported incidence of 15–30 cases per year per 100,000 [1,2]. The age of onset is typically at >50 years with a peak of incidence in the seventh decade of life [3]. As with most immune-mediated rheumatologic disorders, there is a higher preponderance in women [4]. The pathophysiology is thought to be antigen-driven with the activation of T cell and cytokines. Activated T cells stimulate the release of interferon-γ. This ultimately causes the differentiation and migration of macrophages into giant cells. Further release of cytokines IL-1 and IL-6 within the media and intima produce reactive oxygen species and metalloproteinases that cause arterial injury within the medium- and large-sized vessel wall. The subsequent degradation of the lamina results in luminal occlusion [3]. Common clinical manifestations include new onset headache, jaw claudication, fever, weight loss, polymyalgia rheumatica, and acute visual deficits. Untreated GCA can progress to affect the arteries supplying the eye, resulting in vision loss in up to 20% of cases [5].

The superficial temporal artery is commonly affected, making it ideal for biopsy sampling [6]. Temporal artery biopsy (TAB) has been considered the gold standard for diagnosis for a long time, but negative biopsy results can be seen in up to half of cases due to non-involvement of the temporal artery or skip lesions [7]. Advances in technology have made it possible to use ultrasound (US) to aid in the diagnosis of GCA, but due to user inter-variability and differences in centre expertise, temporal artery biopsy is still frequently used as the gold standard test [8]. More recently, other imaging modalities such as MRI, CT, and PET-CT are becoming increasingly available with higher sensitivities than biopsy alone [9]. One study of 64 patients demonstrated a negative predictive value of 98% using PET-CT [10]. However, these newer modalities are expensive, not standardized for use, and there is controversy with regard to their being the most effective choice [9].

The American College of Rheumatology (ACR) criteria, proposed in 1990, score patients out of five categories, including a positive temporal artery biopsy with a score of 3 or more considered diagnostic for GCA (see Table 1) [11]. The ACR criteria have limited benefit in risk stratifying patients pre-biopsy. The limitation of the ACR criteria is obvious when we assess the very elderly population group where an ESR > 50 can be a normal finding [12]. One response to this limitation was a revised ACR criteria (rACR) proposed in 2016 that utilizes an 11-point scoring system. In this system, a diagnosis of GCA can be established with a score of 3 points or more provided all the entry criteria are met and with at least 1 point in domain I (see Table 2) [12]. A clinical management tool has been proposed for the rACR that allows for the possibility of risk stratification and potential diagnosis pre-biopsy (see Table 3) [13].

## 2. Materials and Methods

We conducted a retrospective single centre cohort study of 59 patients undergoing TAB over a 5-year period in a tertiary referral centre from 2013 to 2017. The patients were referred by their rheumatology and medical teams. All TABs were performed under local anaesthetic. Approval was granted by the Beaumont Hospital Audit and Research department. Patient data were collected from the Irish Hospital Inpatient Enquiry (HIPE) database and paper and electronic patient charts. Patient data included age, demographics, clinical features, and biochemical results. Patients with incomplete or missing data were excluded.

The ACR and the rACR scores were compiled for all patients both pre-biopsy and post-biopsy and a comparison was made between the ACR and rACR in their predictive values. The performance of “pre-test ACR”, “ACR”, “pre-test rACR”, and “rACR” scores were investigated using receiver operating characteristics (ROC) analysis and using biopsy results to determine the presence or absence of disease. The area under the curve (AUC) indicates the performance of predicting the biopsy result, where 1.0 = perfect, 0.9 = excellent, 0.8 = good, 0.7 = fair, 0.6 = poor, and <0.5 = useless.

Additionally, sensitivity, specificity, positive predictive value (PPV), and negative predictive value (NPV) were calculated for different threshold scores assessed against the biopsy results. The software R version 3.6.1 was used to perform the data analysis.

## 3. Results

Fifty-nine patients underwent a TAB in the 5-year period from 2013 to 2017. Six patients were excluded in our study due to incomplete or missing data. Of the 53 considered in our study, there were 25 males and 28 females with an average age of 75. The age range was 47 to 93 and the median age was 76. All procedures were done as day case procedures under local anaesthetic with a sterile technique employed. Attempts were made to obtain between 1 and 2 cm in length of biopsy sample where possible. In total there were 11 (20%) positive biopsies.

The ACR criteria were used to calculate the post biopsy scores for all patients. A total of 17 scored in the low-risk category of <3 and 36 scored in the high-risk category of 3–5. All patients with a positive biopsy scored in the high-risk category. The male to female ratio in the low-risk group was 12:5 and in the high-risk group was 13:23.

The ACR criteria were used to compile the ACR score pre biopsy. A total of 19 patients scored in the low-risk group and 34 scored in the high-risk group. Two of the patients who scored as low-risk pre-biopsy were confirmed to have GCA upon biopsy and therefore could potentially be missed if a biopsy was not possible (see Table 4).

The data was then analysed using the rACR criteria. Two patients were excluded as they did not meet the inclusion criteria. A further six patients were excluded as they did not meet the domain I criteria. Therefore, 45 patients were analysed using the rACR scoring system and had their risk stratified.

In the post-biopsy analysis for the rACR, fourteen patients scored as low risk (≤2), nineteen patients scored as intermediate risk (3–4), and twelve patients scored as high risk (≥5). The male to female ratio in each category was 4:3, 11:8, and 1:2, respectively. In the high-risk group, the male to female ratio approached what is expected from the literature when compared to the ratio in the low- and intermediate-risk group. Nine patients with a positive biopsy scored in the high-risk group and two with a positive biopsy scored in the intermediate-risk group.

The pre-biopsy rACR scores were then compiled for our patients. Sixteen scored as low risk, twenty-two scored as intermediate risk, and seven scored as high risk. Four of the positive biopsies were in the high-risk category, five were in the intermediate-risk category, and two were in the low-risk category. Therefore, two patients who scored as low risk could potentially have missed their diagnosis if a TAB was not performed. However, seven patients were categorized as high-risk pre-biopsy (see Table 5).

**Figure 1:**
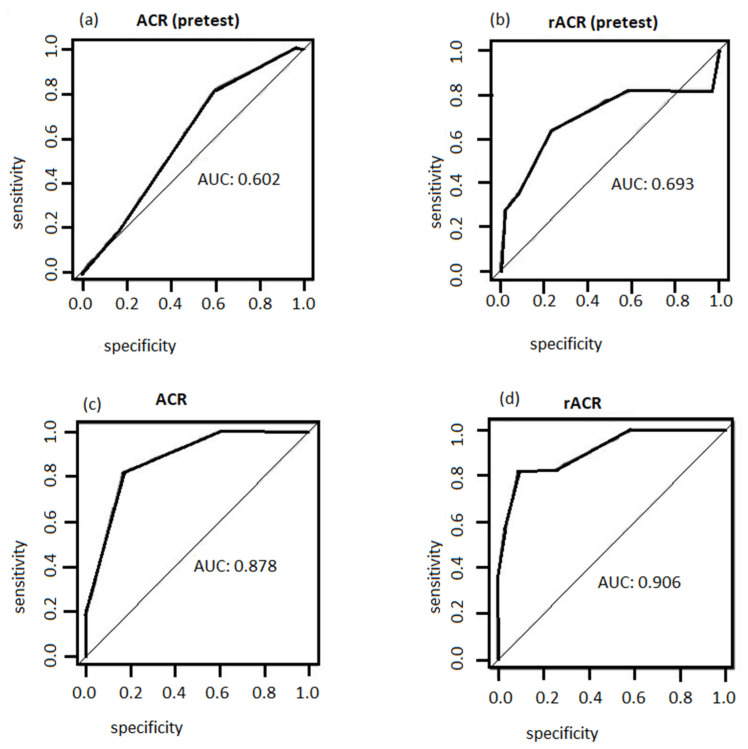
Receiver operating characteristics analysis curves for pre-test ACR (**a**), pre-test rACR (**b**), ACR (**c**), and rACR (**d**) scores and TAB results.

The ROC analyses for the four tests are shown in Figure 1. The rACR test appeared to perform the best to identify the disease as the AUC = 0.9 (excellent), while pretest ACR performed poorly as the AUC = 0.602 (poor). The ACR with AUC = 0.87 (good) outperformed the pretest rACR AUC = 0.69 (fair), but the result showed that the pretest rACR can be used as a good initial diagnostic test before performing a biopsy.

Table 6, Table 7, Table 8 and Table 9 present a summary of the performances of threshold scores for the four tests. For all the tests, lower score cut-off resulted in a high sensitivity (high rate of false positives), a low specificity, and a low PPV. For the pre-test ACR, a threshold of 3 identified 82% of the cases, with a probability of a positive test of 31%. However, the specificity was low, indicating that the test will label many healthy people as having the disease; these patients will therefore benefit from having TAB performed. On the other hand, a score of ≥5 was 91% specific with a PPV and NPV again suggesting that these patients can potentially avoid a biopsy.

The optimal score for ACR is 3–4. Comparing this with rACR, the optimal cut-off value appears to lie within 3–5. A cut-off score of 3 has similar result to the ACR test. A cut-off score of 4 and 5 have the same sensitivity but a score of 5 has a higher sensitivity and a higher PPV. In fact, a cut-off score of 5 is optimal as it has the best trade-off between sensitivity and specificity with a high PPV and NPV. This analysis further supports the advantage of using rACR over ACR, as per Table 3.

## 4. Discussion

The ACR criteria have previously been proposed as a pre biopsy tool to help minimize unnecessary TAB in patients with a score of 3 or more [14]. Although TAB is generally considered a safe procedure, a number of complications can occur from performing this surgical intervention, including bleeding, hematoma, infection, and damage to the facial nerve [15,16,17]. In one study of 111 patients, it was found that 19 of these patients had met the criteria for a GCA diagnosis based on the ACR criteria and could have avoided surgery [18]. On the other hand, a meta-regression in a recent systematic review showed no significant impact of scoring systems on positive TAB yield [19]. Our series demonstrates that the ACR criteria have a limited role in risk stratifying patients based on score; however, 34 of 53 patients had a diagnostic score for GCA and could have potentially avoided surgery.

The more recently proposed rACR criteria have several advantages over the ACR criteria as a pre-biopsy diagnostic tool in that it uses an 11-point scoring system based on clinical and biochemical features [13]. In our case, seven patients scored as high risk and could have avoided biopsy while those in the intermediate risk would benefit from a biopsy. Sait et al., based on their study of 42 TABs, proposed that patients with a score of ≤2 would not need a biopsy or steroid treatment due to their low risk (see Table 3) [13]. Our study, however, suggests that a temporal artery biopsy would be necessary in the low-risk group due to the low specificity, as 2 of 16 will go on to have a positive biopsy result and therefore a diagnosis of GCA. Therefore, we propose that the rACR tool should only be used to prevent biopsy in the high-risk group.

A more recently proposed set of ACR diagnostic criteria has been drafted that uses multiple categories for scoring the risk. This scoring system utilizes ultrasound imaging as an adjunct to compile the total score. While ultrasound imaging is being used in some centres, it is worth noting that the newly published 2021 ACR guidelines still recommend using TAB over temporal artery ultrasound [20,21].

The ROC analysis performed on our data suggests that the rACR scoring system has a better performance when compared to ACR, with a better sensitivity–specificity trade-off and a higher ROC score. For a cut-off score of 5 for the pre-biopsy rACR, we reached a sensitivity and specificity of 82% and 91%, respectively. This is comparable to US imaging, where the reported sensitivity and specificity is 77% and 96% [22].

The strength of this study is the thoroughness of the data, with only 6 of 59 patients excluded due to missing data, and the detailed analysis. The limitations of this study are that it is a single centre study with a small sample size. Furthermore, we did not look at the temporality of the onset of symptoms to the time of surgery. It has been suggested that a temporal artery biopsy should be performed early post-commencement of steroid therapy to give a higher positive yield [13]. However, positive biopsies have been reported even at 4–6 weeks post-commencement of steroid therapy [23,24,25]. Furthermore, it is important to note that there is evidence that the diagnostic yield of a positive biopsy is lower in patients with predominantly large vessel GCA compared to cranial GCA. [26]

While we feel that the rACR is a useful tool that can be used to minimize unnecessary surgery, caution must be taken when utilizing it for the diagnosis of relapse. The 2018 European League Against Rheumatism (EULAR) guidelines recommend initiating steroid treatment to the last effective dose even with minor relapse (i.e., non-ischemic manifestations of GCA) [24]. Furthermore, in our centre, referral for a biopsy was invariably made by a rheumatologist or by a medical team with consult from a rheumatologist. This is in keeping with the 2018 EULAR recommendations [24]. We therefore suggest that the medical and rheumatology specialists familiarize themselves with this tool and employ it prior to making a surgical referral for biopsy.

## 5. Conclusions

This study demonstrates that while the 1990 ACR criteria for GCA have limited benefit in risk stratification in avoiding TAB, the rACR scoring system may be a more useful tool in categorizing patients into low, intermediate, and high risk of GCA pre-biopsy. Performing TAB is of benefit in the low- and intermediate-risk groups as a biopsy can alter the risk category of patients. Surgery can potentially be avoided in patients in the high-risk group, as a positive biopsy does not alter risk category. Moreover, ROC analysis indicates that rACR is superior to ACR as a diagnostic test.

## Figures and Tables

**Table 1:** ACR criteria.

Criteria
Age > 50
New onset localized headache
Tenderness over temporal artery/ decreased pulse
ESR > 50 mm/h
Positive biopsy (Biopsy specimen showing vasculitis, predominance of mononuclear cell infiltration, granulomatous inflammation, with multi nucleated cells.)

**Table 2:** rACR criteria ^a^.

Entry Criteria	
Age > 50	
Absence of exclusion criteria ^b^	
**Domain I Criteria**	
New onset localized headache ^c^	1 point
Sudden onset visual disturbance ^c^	1 point
Polymyalgia rheumatica	2 points
Jaw claudication ^c^	1 point
Abnormal temporal artery ^d^	Max 2 points
**Domain II Criteria**	
Unexplained fever/ anaemia	1 point
ESR ≥ 50 mm/h ^e^	1 point
Compatible pathology ^f^	Max 2 points

^a^ In the presence of 3 points or more out of 11 with at least 1 point belonging to domain I along with all entry criteria, the diagnosis of giant cell arteritis can be established. ^b^ Exclusion criteria include: ear, nose, throat, and eye inflammation, kidney, skin and peripheral nervous system involvement, lung infiltration, lymphadenopathies, stiff neck, and digital gangrene or ulceration. ^c^ No other aetiologies can better explain any one of the criteria. ^d^ Enlarged and/or pulseless temporal artery: 1 point; tender temporal artery: 1 point. ^e^ It must be ignored in the presence of PMR. ^f.^ Vascular and/or perivascular fibrinoid necrosis along with leukocyte infiltration: 1 point and granuloma: 1 point.

**Table 3:** Clinical tool for rACR criteria.

rACR Score	Comments	Management
≤2	Unlikely GCA	No TAB or steroids
3 and 4	Requires confirmation	Perform TAB
≥5	Likely GCA	No TAB, continue steroids

**Table 4:** ACR scores (n = 53).

Post-Biopsy Score	
Low-risk score < 3	High-risk score 3–5
17	36 (11 biopsies proven)
**Pre-Biopsy score**	
Score < 3	Score 3–5
19 (2 biopsy proven)	34 (9 biopsies proven)

**Table 5:** rACR (n = 45).

Post-Biopsy Score		
Score ≤ 2	Score 3–4	Score ≥ 5
14	19 (2 biopsies proven)	12 (9 biopsies proven)
**Pre-Biopsy Score**		
Score ≤ 2 (Low risk)	Score 3–4 (intermediate risk)	Score ≥ 5 (high risk)
16 (2 biopsies proven)	22 (5 biopsies proven)	7 (4 biopsies proven)

**Table 6:** Performance scores for ACR (pre-test) and TAB.

ACR Score (x/4)	Sensitivity	Specificity	PPV	NPV
2	100%	5%	22%	100%
3	82%	40%	26%	89%
4	18%	83%	22%	80%

**Table 7:** Performance scores for rACR (pre-test) and TAB.

rACR Score (x/9)	Sensitivity	Specificity	PPV	NPV
2	82%	3%	21%	33%
3	82%	41%	31%	88%
4	64%	76%	47%	87%
5	36%	91%	57%	82%
6	27%	97%	75%	80%

**Table 8:** Performance scores for ACR and TAB.

ACR Score (x/4)	Sensitivity	Specificity	PPV	NPV
2	100%	5%	22%	100%
3	100%	40%	31%	100%
4	82%	83%	56%	95%

**Table 9:** Performance scores for rACR and TAB.

rACR Score (x/9)	Sensitivity	Specificity	PPV	NPV
2	100%	3%	25%	100%
3	100%	41%	35%	100%
4	82%	76%	53%	93%
5	82%	91%	75%	94%
6	55%	97%	86%	87%

## Data Availability

Data available are upon request and are not available publicly due to restrictions with GDPR audit policies.
